# Appetitive traits associated with higher and lower body mass index: evaluating the validity of the adult eating behaviour questionnaire in an Australian sample

**DOI:** 10.1186/s12966-017-0587-7

**Published:** 2017-09-22

**Authors:** Kimberley M. Mallan, Alison Fildes, Xochitl de la Piedad Garcia, Jayne Drzezdzon, Matthew Sampson, Clare Llewellyn

**Affiliations:** 1 School of Psychology, Australian Catholic University, 1100 Nudgee Road, Banyo, QLD 4014 Australia; 2 School of Exercise and Nutrition Sciences, Queensland University of Technology, 60 Musk Avenue, Kelvin Grove, QLD 4059 Australia; 3 School of Psychology, University of Leeds, Woodhouse Lane, Leeds, LS2 9JT UK; 4 Department of Behavioural Science and Health, University College London, Gower St, London, WC1E 6BT UK

**Keywords:** Appetitive traits, Eating behaviour, Appetite, Obesity, Adults, Confirmatory factor analysis

## Abstract

**Background:**

The aims of this study were to evaluate the factor structure of the newly developed Adult Eating Behaviour Questionnaire (AEBQ) (Hunot et al., Appetite 105:356-63, 2016) in an Australian sample, and examine associations between the four food approach and four food avoidance appetitive traits with body mass index (BMI).

**Methods:**

Participants (*N* = 998) recruited between May and October 2016 via a university research participation scheme and online social network sites completed an online version of the AEBQ and self-reported demographic and anthropometric data. Of the sample, 84.8% were females, 29.6% had completed a university degree and the overall mean age was 24.32 years (SD = 8.32). Confirmatory factor analysis (CFA) was used to test three alternative factor structures (derived from issues raised in the original development study): the original 8 factor model, a 7 factor model with Food Responsiveness and Hunger scales combined, and a 7 factor model with the Hunger scale removed.

**Results:**

The CFA revealed that the original 8 factor model was a better fit to the data than the 7 factor model in which Food Responsiveness and Hunger scales were combined. However, while reliability estimates for 7 of the 8 scales were good (Cronbach’s α between 0.70-0.86), the reliability of the Hunger scale was modest (0.67) and dropping this factor resulted in a good fitting model. All food avoidance scales (except Food Fussiness) were negatively associated with body mass index (BMI) whereas Emotional Overeating was the only food approach scale positively associated with BMI.

**Conclusions:**

The study supports the use of the AEBQ as a reliable and valid measure of food approach and avoidance appetitive traits in adults. Longitudinal studies that examine continuity and stability of appetitive traits across the lifespan will be facilitated by the addition of this measurement tool to the literature.

## Background

At a time when obesity has become a public health crisis affecting children and adults across the globe, there has never been more focus on our eating habits. The current food environment and sedentary lifestyles, dubbed ‘obesogenic’, may to some extent explain the global rise in the prevalence of obesity [1]. However a potential explanation as to why some individuals maintain a healthy weight whilst others become obese has been provided by the behavioural susceptibility model in which inherited traits may interact with these environmental factors to increase or attenuate obesity risk [2]. In particular, inherited differences in eating behaviours – or ‘appetitive traits’ – that make some individuals more susceptible to *overeating*, may account for differences in energy intake and ultimately weight status from early in life [3–5].

Individual differences in appetitive traits such as heightened responsiveness to food cues (e.g., eating in response to the sight, smell, or taste of palatable foods) and emotional overeating (e.g., eating more in response to negative emotions) have consistently been associated with excess energy intake and higher weight [4, 6]. From birth, differences in ‘appetite’ have been prospectively associated with rate of weight gain [7–9]. In children, the study of the relationship between both ‘food approach’ (e.g., Food Responsiveness, Enjoyment of Food) and ‘food avoidance’ (e.g., Satiety Responsiveness, Food Fussiness) appetitive traits and body mass index (BMI) [10–13], observed eating behaviour/intake [5, 14], food preferences [15], and parental feeding practices [16–19] has been facilitated by the widely used Children’s Eating Behaviour Questionnaire (CEBQ) [20].

The CEBQ is a parent-report instrument that measures 8 appetitive traits. In multiple samples across the world the CEBQ has shown good reliability and a robust factor structure [11, 20–22]. In 2011 an infant version of the CEBQ – the Baby Eating Behaviour Questionnaire (BEBQ) [23] was developed to assess equivalent food approach and food avoidance appetitive traits during the period of exclusive milk feeding. The BEBQ has shown good reliability, robust factor structure and associations with weight status in multiple samples [23, 24].

To date the measurement of eating behaviours in adults has tended to focus heavily on eating behaviours specifically related to disordered eating and/or obesity risk. The two most prominent and extensively validated questionnaires in the field are the Three-factor Eating Questionnaire [25] which measures constructs of dietary restraint, disinhibition and hunger, and the Dutch Eating Behaviour Questionnaire [6, 26] which also measures three constructs: restraint, emotional eating and external eating. However, these measurement tools for use in adult samples have not included items that reflect appetitive traits such as satiety responsiveness and slowness in eating which may protect against excess energy intake and overweight. Similarly, there has been little research in adults on picky or fussy eating, and when fussy eating has been assessed it tends to have been treated as a categorical variable (i.e., picky vs not picky) [27]. The recently developed Adult Eating Behaviour Questionnaire (AEBQ) [28] was specifically designed to address these gaps in the literature. The AEBQ includes 8 scales encompassing both food approach (Hunger, Food Responsiveness, Emotional Over-eating and Enjoyment of Food) and avoidance appetitive traits (Satiety Responsiveness, Food Fussiness, Emotional Under-eating and Slowness in Eating).

The AEBQ was developed based on the CEBQ/BEBQ with the intention that it would allow for future studies to examine the continuity and stability of appetitive traits from infancy into adulthood using similar measurement tools. In a community sample of 954 UK adults the factor structure of the AEBQ was evaluated using confirmatory factor analysis and the mean scale scores were correlated with self-reported BMI. An 8 factor model showed a good fit to the data and, as expected, the food approach scales (except Hunger) and the food avoidance scales (except Food Fussiness) were significantly associated with lower and higher BMI respectively [28].

To our knowledge the factor structure of the AEBQ has not yet been replicated and has not been validated in a sample outside the UK. As with all new measurement tools it is important to replicate the factor structure in a new sample and to examine aspects of validity, such as associations between subscales and BMI. Furthermore, the authors of the AEBQ indicated some reservation over the novel ‘Hunger’ scale (unlike the other scales, this was an entirely novel scale and is not included in the CEBQ); specifically whether the items on this scale should be combined with or remain separate from the Food Responsiveness items, or whether the scale should be retained at all [28]. The present study aimed to examine the reliability and validity of the AEBQ in an Australian sample by evaluating: 1) the psychometric properties of the scales and the overall factor structure using the gold-standard method of confirmatory factor analysis; and 2) associations between the scales and BMI to establish construct validity. It was hypothesised that the food approach scales would be associated with higher BMI whereas the food avoidance scales would be associated with lower BMI.

## Method

### Study design and participants

In this cross-sectional study participants were invited to complete an anonymous online survey via an online research participation site whereby students studying psychology at Australian Catholic University could voluntarily participate in research studies and through a social networking site (i.e., Facebook). Of the total sample (*N* = 998), 313 were recruited via the university psychology research participation site and 590 were recruited via Facebook. Due to the nature of the sampling approach used a response rate could not be estimated. Eligibility criteria for participation were: English speaking, no current diagnosed psychological disorder and no current or history of diagnosed eating disorder(s). Participants recruited through the university research site gained credit towards a psychology unit. All other participants were eligible to enter a draw to win a set of headphones (valued at approximately AUD250) or one of 10 shopping vouchers (valued at AUD20 each).

### Measures

#### Demographics

Participants self-reported age, gender, highest level of education completed (primary, secondary, post-secondary school certificate/diploma, undergraduate university degree, postgraduate university degree), and ethnic background.

#### Appetitive traits

Appetitive traits were assessed via the AEBQ [28]. The 35 item questionnaire comprises 4 ‘food approach’ subscales: Hunger (5 items; e.g. *I often feel hungry*); Food Responsiveness (4 items; e.g. *I am always thinking about food*); Emotional Over-eating (5 items; e.g. *I eat more when I’m upset*); Enjoyment of Food (3 items; e.g. *I enjoy eating*), and four ‘food avoidance’ subscales: Satiety Responsiveness (3 items; e.g. *I get full up easily*); Emotional Under-eating (5 items; e.g. *I eat less when I’m worried*); Food Fussiness (5 items including 3 reverse coded items; e.g. *I refuse new foods at first*), and Slowness in Eating (4 items including 1 reference coded items; e.g. *I eat slowly*). Item responses were recorded on a 5-point Likert scale ranging from ‘Strongly Disagree’ to ‘Strongly Agree’. Mean scores were calculated for each subscale.

#### Anthropometrics

Body mass index (kg/m^2^) was calculated based on self-reported height and weight data.

### Statistical analysis

Descriptive statistics and reliability estimates for the AEBQ scales were conducted in IBM SPSS Version 22. Similarly, relationships between AEBQ scales (unweighted means) and BMI were examined via Pearson’s correlations and multivariable linear regression analyses were used to control for gender, age and sample (university vs online).

The sample had 0.33% missing data on the AEBQ items overall and ≤1.00% missing data on each of the 35 items. Thus, in order to avoid deletion of cases, missing data were imputed using Expectation Maximisation (EM) imputation in SPSS Version 22 prior to further analysis. Confirmatory factor analysis using structural equation modelling (IBM AMOS V.22) tested the fit to the data of three alternate models based on the findings of Hunot et al. [28]. Model 1 included all 35 items loading onto the 8 original factors of the AEBQ. Model 2 included all 35 items loading onto 7 factors, with the Hunger and Food Responsiveness items loading onto a single factor. Finally, Model 3 excluded the Hunger factor (i.e. only 30 items loading onto 7 factors were included). In all cases specifications for the models included: correlated factors, uncorrelated error variances and fixing the variance of the first item on each factor to 1. The hypothesised models were un-identified. Based on recommendations [29, 30], the acceptability of model fit was evaluated against achievement of the following criteria: Tucker Lewis Index (TLI) approaching 0.90; Comparative Fit Index (CFI) ideally >0.90, and Root Mean Square Error of Approximation (RMSEA) ideally ≤0.06. Item-factor loadings, factor variance and item variance were also considered when evaluating model fit. Parsimony of alternate models (1 and 2 only) was assessed using Akaike’s Information Criteria (AIC) whereby small values indicated a more parsimonious model. Given that the primary aim of the study was to compare these three alternate factor structures of the AEBQ, modifications (e.g. adding error covariances) to improve model fit were not considered.

## Results

The characteristics of the sample are presented in Table 1. The sample was over-represented by young, Caucasian females.

**Table 1:** Characteristics of *N* = 998 participants recruited via a university research scheme (*n* = 408) and via social media (*n* = 590)

Characteristics	Mean ± standard deviation or % (n)
Age (years) (*n* = 893)	24.32 ± 8.32
Gender (female) (*n* = 996)	84.8% (845)
BMI (kg/m^2^)^a^ (*n* = 983)	24.90 ± 5.60
Highest level of education (*n* = 995)	
Primary school	1.2 (12)
Secondary school	44.0 (438)
Certificate/diploma	25.1 (250)
Undergraduate university degree	24.2 (241)
Postgraduate university degree	5.4 (54)
Ethnicity (n = 996)	
Caucasian	80.9 (806)
Asian	6.3 (63)
Hispanic	1.6 (16)
African	1.1 (11)
Aboriginal or Torres Strait Islander	1.6 (16)
Pacific Islander	1.8 (18)
Other	6.6 (66)

^a^based on self-reported height and weight data

Reliability estimates and mean scale scores for the AEBQ are presented in Table 2 alongside the corresponding values from the Hunot et al. study [28] (includes unpublished data obtained directly from the author). In the present sample Cronbach’s alpha values for all scales except for Hunger were ≥.70, and were generally comparable to those values from the original UK sample. Examination of mean scale scores indicated relatively higher means for food approach scales compared to food avoidance scales (Table 2) within the present sample. Numerically higher mean scores were observed for the food approach scales in the present sample compared to the original UK sample (Food Responsiveness +.51, Enjoyment of Food + .37 and Hunger + .30, Emotional overeating +.22). Differences in mean scores were negligible between the samples for the food avoidance scales.

**Table 2:** Descriptive statistics (mean ± standard deviation) and internal consistency estimates (Cronbach’s α) for the 8 factor Adult Eating Behaviour Questionnaire (AEBQ) in the present Australian sample (N = 998; mean age = 24 ± 8; 84.8% female) and in the original validation sample of British adults (*N* = 954; mean age = 44 ± 13; 57.3% female)

	Australian sample (present study)		UK sample (Hunot et al. study [28]^a^)	
AEBQ subscale	Cronbach’s α	Mean ± SD	Cronbach’s α	Mean ± SD
Food approach subscales				
Hunger	.67	3.22 ± 0.74	0.75	2.92 ± 0.78
Food responsiveness	.70	3.49 ± 0.74	0.75	2.98 ± 0.78
Emotional over-eating	.85	2.96 ± 0.91	0.90	2.74 ± 0.98
Enjoyment of food	.85	4.37 ± 0.69	0.86	4.00 ± 0.74
Food avoidance subscales				
Satiety responsiveness	.75	2.76 ± 0.83	0.75	2.61 ± 0.81
Emotional under-eating	.87	2.96 ± 0.89	0.90	2.83 ± 0.92
Food fussiness	.87	2.26 ± 0.83	0.88	2.29 ± 0.84
Slowness in eating	.86	2.74 ± 1.00	0.88	2.62 ± 0.97

^a^includes unpublished data

Fit indices for the three alternate models of the AEBQ are presented in Table 3. For all models all factor variances were significant (*p* < .001), all factor-item loadings were above .300 and significant (p < .001), and all item squared multiple correlations were above .1. The original 35-item, 8 factor model Model 1) showed good fit to the data and was clearly superior to the 35-item, 7 factor model (Model 2) according to all fit indices considered and was a more parsimonious model according to the AIC values (Table 3). The 30 item, 7 factor Model (Model 3), although not directly comparable to Models 1 and 2 due to a different number of items, also showed good fit overall (see Table 3). These results provide support for the original 8 factor model (Model 1) or a 7 factor model that excludes the Hunger factor/items entirely (Model 3).

**Table 3:** Fit indices of three models of the Adult Eating Behaviour Questionnaire evaluated via confirmatory factor analysis in a sample of 998 Australian adults

Model	Items	Factors	χ^2^(df)	χ^2^/df	TLI	CFI	RMSEA	AIC
Model 1	35	8 (H and FR items load on separate factors)	2059.853 (532)	3.872	.894	.905	.057	2325.853
Model 2	35	7 (H and FR items load on combined factor)	2232.461 (539)	4.142	.884	.895	.056	2484.461
Model 3	30	7 (H items/factor deleted)	1652.769 (384)	4.304	.914	.914	.058	n/a

*H* Hunger scale, *FR* Food Responsiveness scale, *χ* ^*2*^ */df* normed chi-square, *TLI* Tucker Lewis Index, *CFI* Comparative Fit Index, *RMSEA* Root Mean Square Error of Approximation, *AIC* Akaike’s Information Criteria

As expected the food approach scales were positively inter-correlated and were generally negatively correlated with the food avoidance scales (Table 4). Unexpectedly there was a positive correlation between Hunger and Emotional Under-eating. The food avoidance scales were also positively inter-correlated however Food Fussiness was not significantly related to Emotional Undereating (Table 4). Associations with self-reported BMI are also presented in Table 4 (unadjusted and adjusted for age, gender and sample). As expected Emotional Over-eating was associated with a higher BMI but contrary to predictions the other food approach scales were not: Food Responsiveness and Enjoyment of Food were not associated with BMI and Hunger was significantly associated with a lower BMI. In line with expectations, all food avoidance scales, except for Food Fussiness, were associated with a lower BMI.

**Table 4:** Pearson’s correlations between the 8 Adult Eating Behaviour Questionnaire subscales (*N* = 998) and with self-reported BMI (*n* = 983) in an Australian sample

	Correlations									
	H	FR	EOE	EF	SR	EUE	FF	SE	BMI	
									Unadjusted (r)	Adjusted ^a^ (β)
Food approach subscales										
Hunger	1	.52**	.27**	.33**	−.093**	.099*	>.001	−.023	−.16**	−.13**
Food responsiveness		1	.38**	.51**	−.26**	−.075*	−.054	−.17**	−.081*	−.024
Emotional over-eating			1	.18**	−.091*	−.52**	.069*	−.13**	.14**	.15**
Enjoyment of food				1	−.25**	−.056	−.21**	−.42**	−.022	.011
Food avoidance subscales										
Satiety responsiveness					1	.25**	.27**	.48**	−.17**	−.15**
Emotional under-eating						1	−.026	.19**	.007	.030
Food fussiness							1	.087*	−.15**	−.11**
Slowness in eating								1	−.16**	−.13**

^a^Analyses adjusted for gender, age and sample (university or online); *n* = 883 due to missing data; *p < .05, **p < .001

## Discussion

The purpose of this study was to evaluate the reliability and validity of the AEBQ using CFA, and examine associations with BMI in an Australian sample. The AEBQ was designed to measure 4 food approach and 4 food avoidance appetitive traits in human adults. The results of the CFA provided support for the 8 factor structure of the AEBQ proposed by Hunot et al. [28] over a 7 factor version in which the Hunger and Food Responsiveness items were combined. Correlations between subscales and internal reliability estimates added further support to the utility of the questionnaire. Consistent with the hypothesised pattern of associations and the findings of Hunot et al. [28], all food avoidance scales, except for Food Fussiness, were associated with a lower BMI and Emotional Over-eating was associated with higher BMI. However, inconsistent with predictions, the Hunger scale was negatively associated with BMI and the Food Responsiveness and Enjoyment of Food scales showed no association with BMI in this sample.

Three alternative factor structures of the AEBQ were evaluated in this study and the results favoured the original 8 factor model over a 7 factor model combining Hunger and Food Responsiveness. However, it was suggested by Hunot et al. [28] that the Hunger scale may be removed altogether in future iterations of the questionnaire as it failed to show the expected positive association with BMI in the original study. The utility of the scale was also questioned by the authors because individuals differ in how they perceive and interpret physical hunger [6] and because it may be affected by other aspects of eating regulation such as dietary restraint [31]. The present results provide some support for the suggestion that the Hunger scale may require further investigation to determine its value to the AEBQ. Firstly, the reliability of the Hunger scale fell below .70, substantially lower than in the original sample (0.67 vs 0.75) [28] and deletion of items (with the lowest loading in the CFA) did not improve reliability. Secondly, combining the Hunger and Food Responsiveness items did not improve the structure of the AEBQ and resulted in a less parsimonious and worse-fitting model. Thirdly, removal of the Hunger scale entirely resulted in a model that showed a good fit to the data. Finally, as will be discussed further below, the Hunger scale showed a number of unexpected correlations with another scale of the AEBQ and with BMI.

Generally, the patterns of associations between the scales of the AEBQ were similar to those reported in the original validation paper [28]. The food approach scales were positively related to one another and tended to be negatively correlated with the food avoidance scales. Of note, however, was the positive association between Hunger and the food avoidance trait Emotional Under-eating, a finding which was also observed in the original study [28] and further clouds the interpretation of the Hunger scale. As expected the food avoidance scales were positively related to one another; although Food Fussiness was not significantly related to Emotional Under-eating and was only weakly associated with Slowness in Eating. These patterns of association seem to indicate that Food Fussiness is qualitatively distinct from the other food avoidance traits. At face value, it seems logical that Food Fussiness reflects selectivity in food choice whereas the other food avoidance scales reflect smaller appetite and heightened sensitivity to satiety cues. Nevertheless, longitudinal studies of the relationship between all of the appetitive traits and BMI are needed to assess direction of effects. While Food Fussiness was not associated with BMI in this sample, it may be maladaptive in terms of achieving adequate dietary variety and diversity [32]. Examining associations between the AEBQ scales and measures of food preferences, intake or diet quality will help to test these predictions.

As hypothesised, the food avoidance traits Satiety Responsiveness, Emotional Under-eating and Slowness in Eating, were all associated with lower BMI; indicating that these scales capture dimensions of appetite. Food Fussiness was unrelated to BMI further supporting the suggestion that this scale reflects a qualitatively different food avoidance trait and is not protective against excess weight gain in an obesogenic environment. Whilst there is some evidence that Food Fussiness may be negatively associated with BMI in children [21, 33, 34], fussiness in children has been associated with higher intake of sweetened foods [35] and has been suggested as a risk factor for excessive weight gain in the longer term if intake of foods such as fruits and vegetables are replaced with intake of energy-dense, highly palatable foods (high in sugar, fat and/or salt) [36]. In adults, the relationship between fussy eating and BMI is also unclear. In a study of young adults no differences in BMI between ‘selective eaters’ and ‘non-selective eaters’ was found [37]. In contrast, findings from both human and animal studies of obesity have suggested that fussiness may be higher in obese individuals and be associated with excess energy intake depending on the availability of highly palatable foods [38, 39].

The relationships between the food approach traits and BMI were less straightforward. While Emotional Over-eating was associated with significantly higher BMI in this sample, the other food approach scales were not significantly associated with BMI. In fact, both Hunger and Food Responsiveness showed small but significant correlations with lower BMI in the unadjusted analyses. After adjusting for gender, age and sample the association between BMI and Food Responsiveness became non-significant whereas the association with Hunger remained. These results are not consistent with those of Hunot et al. [28]. However, it is worth considering that the positive and significant (*p* < .05) correlations between self-reported BMI and Food Responsiveness and Enjoyment of Food scales in their study were very small (*r* = 0.071 and 0.067, respectively) and there was actually a very small and nonsignificant negative correlation between BMI and the Hunger scale (*r* = −.028, *p* > .05). Interpretation of these somewhat discrepant findings is complicated by the fact that BMI was self-reported in both the present and earlier study, and therefore less reliable than researcher-measured anthropometrics.

Higher scores on the Hunger items (meant to measure physical hunger [28]) and/or Food Responsiveness items of the AEBQ may reflect restrained eating or dieting behaviour in some individuals. This could be particularly relevant for this predominantly young Caucasian female sample. It seems plausible that actively restricting food intake may result in more frequent feelings of hunger and greater responsiveness to the sight and smell of palatable foods. Thus, the Hunger scale in particular may capture the *state* of physical hunger rather than an established appetitive *trait.* There is no Hunger scale included in the CEBQ [20] which means it is not possible to make comparisons with child samples, but the CEBQ Food Responsiveness scale has consistently been associated with both higher weight [20] and increased energy intake measured objectively [14] in children. Thus the absence of a positive association with BMI in the present study is surprising. However, children are unlikely to actively restrict their energy intake, whereas conscious attempts at controlling food intake in order to regulate weight might be responsible for supressing the expected relationship between Food Responsiveness and BMI in the present sample. Interestingly, mean scores on the Hunger and Food Responsiveness scales appeared to be higher in the present sample compared to the older UK sample [28] (mean age = 44, SD = 13) which consisted of a more balanced number of males and females (42.7% male). Basic demographic differences could be responsible for the different findings across the two studies. In our young female sample, many of whom were university students, newly-obtained independence from the family home setting, and associated changes in eating patterns and alcohol consumption, along with peer influences, may impact on appetitive traits – or the perception of one’s own appetitive traits. Further testing of these hypotheses by investigating associations between Hunger and Food Responsiveness and measures of dietary restraint is needed to better understand how these scales should be interpreted.

Despite the novelty of the data presented here and the large sample size there are a number of limitations to consider. The most salient being the over-representation of young Caucasian women in the sample. Whilst gender was adjusted for in the analyses, due to the relatively small number of men, gender differences on scale scores nor potential moderation effects of gender with association between eating behaviours and BMI could not be confidently explored. Future validation of the AEBQ in diverse populations will allow for the broader use of the tool. As indicated, measures of restrained eating [25] or dieting behaviour would be useful to include in future studies to understand better the interaction between the appetitive traits assessed via the AEBQ and other dietary control behaviours that may contribute to energy intake and BMI in adults. Finally, the use of self-reported height and weight to calculate BMI is a limitation and reporting of these anthropometrics may be subject to bias.

## Conclusions

In sum, the current study supports the reliability of the AEBQ as a measure of appetitive traits in adults. The robust factor structure and pattern of associations between the AEBQ scales and BMI lend support to the construct validity of the tool. Future investigation of systematic variation in appetitive traits by factors such as gender and age may give insight into the specific eating behaviours that may make individuals more or less susceptible to excess weight gain throughout adulthood. Furthermore, investigation of the relationship between food approach scales and dieting or restrained eating as well as between Food Fussiness and measures of poor dietary variety is warranted. It is anticipated that the present findings will encourage use of the AEBQ as a convenient and cost-effective self-report tool that will be useful in characterising obesity risk behaviours in adults and will allow researchers to track the development of appetitive traits throughout infancy, childhood and across adulthood by using the BEBQ [23], CEBQ [20] and AEBQ [28], respectively.

## Abbreviations

AEBQ: Adult Eating Behaviour Questionnaire
AIC: Akaike’s Information Criterion
BEBQ: Baby Eating Behaviour Questionnaire
BMI: Body mass index
CEBQ: Children’s Eating Behaviour Questionnaire
CFA: Confirmatory factor analysis
CFI: Comparative fit index
RMSEA: Root Mean Square Error of Approximation
TLI: Tucker Lewis Index
